# Trying to Survive in an Emotional Storm: A Qualitative Framework Analysis of Anonymous Youth Helpline Conversations on Mental Health and Everyday Life

**DOI:** 10.3390/nursrep16080284

**Published:** 2026-08-15

**Authors:** Nina Gårevik, Petra Löfstedt, Lilly Augustine, Lise-Lott Rydström, Maria Jirwe

**Affiliations:** 1 Department of Health Sciences, Swedish Red Cross University, P.O. Box 1059, SE-141 21 Huddinge, Sweden; maria.jirwe@rkh.se; 2 Department of Public Health and Community Medicine, Sahlgrenska Academy, University of Gothenburg, P.O. Box 400, SE-405 30 Göteborg, Sweden; petra.lofstedt@gu.se; 3 CHILD, School of Education and Communication, Jönköping University, P.O. Box 1026, SE-551 11 Jönköping, Sweden; lilly.augustine@ju.se; 4 Department of Neurobiology, Care Sciences and Society (NVS), Karolinska Institutet, Fack 23400, SE-141 83 Huddinge, Sweden; lise-lott.rydstrom@ki.se

**Keywords:** adolescent, mental health, help-seeking behavior, youth helpline, qualitative research, self-determination theory

## Abstract

**Background/Objectives:** Young people’s mental health is increasingly discussed using diagnostic terms. However, limited knowledge exists about how young people describe distress and support needs. This study explored how young people with self-reported functional difficulties described challenges affecting their mental health and everyday lives. **Methods:** A qualitative study was conducted using 137 anonymized chat and text message contacts from BRIS (Children’s Rights in Society), a Swedish youth helpline. Contacts registered within the category “Functional Impairments” were selected through predefined inclusion criteria. The material was analysed in Swedish using Framework Analysis. **Results:** One overarching theme, *Trying to Survive in an Emotional Storm*, was identified, comprising three subthemes: *Social Environment—Between Norm Pressure and the Need for Support*, *A Constant Struggle in the Educational System and Insufficient Parental Support—Between Desire for Help and Barriers to Understanding*. Young people described challenges related to social relationships, school demands, and limited support from adults. Self-reported diagnostic labels, particularly ADHD, were used to explain and communicate distress. **Conclusions:** The findings suggest that distress was closely linked to social and contextual circumstances. For nurses and other mental health professionals, the findings highlight the importance of listening to young people’s narratives and supporting participation, autonomy, and meaning-making.

## 1. Introduction

Young people with long-term health conditions experience not only medical challenges, but also social and psychological difficulties that shape their everyday lives and overall well-being [1]. According to the World Health Organization’s International Classification of Functioning, Disability and Health (ICF), disability arises in the interaction between the individual and their environment, rather than being an attribute of the individual alone. This implies that factors such as lack of support, bullying, and inadequate adaptation in school environments may restrict young people’s participation to a greater extent than the impairment itself [2,3]. From a nursing perspective, particularly in relation to the school nurse’s role, there is a need to recognize how the school environment and access to support influence adolescents’ health, well-being, and participation in everyday life [4].

In this study, functional difficulties refer to self-reported challenges affecting participation in everyday life, regardless of formal diagnosis. These difficulties may be related to neurodevelopmental conditions, other long-term health conditions, or mental health challenges. Disability is understood in line with the ICF framework as arising through the interaction between the individual and the environment.

In recent years, the number of diagnoses among children and adolescents has increased in Europe, particularly in relation to neurodevelopmental conditions [1]. At the same time, concerns have been raised about possible overdiagnosis and the need for more comprehensive forms of support [5,6,7]. Diagnostic categories are not only medical but are also shaped by social and institutional contexts [8,9,10]. For young people, this can influence both identity and opportunities for participation in everyday life [11]. This development remains debated, as some researchers argue that the increase in diagnoses reflects a growing tendency to interpret normal variations in behaviour and development in medical terms, while others emphasize that it represents improved recognition of young people’s needs [12].

According to the Health Behaviour in School-aged Children (HBSC) study, the proportion of 11-, 13-, and 15-year-olds reporting a long-term illness, disability, or medically diagnosed condition increased in Sweden between 2013/14 and 2021/22 [11]. Among boys, the proportion increased in all three age groups, from 21% to 27% among 11-year-olds, from 19% to 29% among 13-year-olds, and from 21% to 30% among 15-year-olds. Among girls, an increase was observed only among 15-year-olds, from 23% to 32% [11]. Children with disabilities also report lower levels of life satisfaction, poorer self-rated health, and higher levels of psychosomatic symptoms compared with their peers without disabilities [13].

Furthermore, self-reported health complaints have increased over time, and many children and adolescents experience recurrent symptoms. Taken together, these findings suggest that both long-term health problems and subjective health complaints have become more common among school-aged children in Sweden [11].

In addition, recurring concerns have been raised in Sweden regarding unequal access to support services for children and adolescents with disabilities. The United Nations Committee on the Rights of the Child has emphasized children’s rights to participation and influence in matters affecting their lives [5,6]. In the present study, participation is understood as young people’s opportunity to express their experiences and to have their perspectives taken seriously in contexts related to their everyday lives and health. Qualitative research enables a deeper understanding of young people’s experiences of mental health and everyday life [14] and is therefore central to the development of relevant nursing care. Therefore, a qualitative approach was chosen to gain a deeper understanding of how young people with self-reported functional difficulties, contacting a national youth helpline due to mental health difficulties, describe and make sense of their situation.

In this study, Self-Determination Theory (SDT) is used as an interpretive framework to explore how the basic psychological needs of autonomy, competence, and relatedness can be understood in relation to young people’s encounters with family, school, and health care services [15,16]. The theory did not guide coding or theme development but was applied during the interpretive phase of the study to support understanding of the findings. Previous research shows that the experience of being listened to is crucial in help-seeking processes, making the contexts in which young people express their experiences, such as chat and telephone interactions, particularly important from a nursing perspective [17]. Online help-seeking has been shown to offer several advantages for young people, including anonymity, immediacy, and the opportunity to communicate with adults in supportive roles, while also functioning as a potential gateway to further support [18].

While qualitative interview studies have provided important insights into young people’s experiences of mental health, such studies are inevitably shaped by the research context, including retrospective reflection and the presence of a researcher. Anonymous youth helpline conversations offer a complementary perspective by capturing how young people describe their feelings, struggles, and everyday experiences in their own words when reaching out for support. This provides access to aspects of their experiences that may not always emerge in formal interviews and enables exploration of how young people themselves understand and make sense of their situation.

Despite extensive research on adolescent mental health, there is still limited knowledge about how young people themselves talk about their difficulties and support needs in naturally occurring anonymous conversations. Analysing such conversations can contribute to a deeper understanding of how young people themselves describe everyday challenges, emotional distress, and support needs in a help-seeking context.

The study is based on data from BRIS (Children’s Rights in Society), a Swedish national support organization providing anonymous support through channels such as chat and telephone [19]. This context allows young people to express their experiences in their own words outside formal healthcare settings, thereby offering valuable insights into how support is experienced and sought. The study was not initially designed to explore specific diagnoses. Nevertheless, neurodevelopmental conditions were prominent in many accounts, reflecting how young people themselves interpret their experiences. Other forms of mental health difficulties, such as anxiety and stress, were also prominent and are here understood as significant in relation to everyday functioning.

The aim of this study is to explore how young people with self-reported functional difficulties who contact a national youth helpline due to challenges affecting their mental health and everyday lives describe and make sense of their situation.

## 2. Materials and Methods

### 2.1. Design and Methodological Approach

This study used a qualitative, exploratory design based on anonymised routine service data from BRIS. Framework Analysis [20,21], a well-established approach within behavioural, caring, and public health sciences, was used as the analytical approach. Framework Analysis was selected because it is well suited to applied health research and enables the integration of predefined analytical interests with themes emerging inductively from the data. The approach was also considered appropriate given the relatively large dataset and the study’s aim of exploring both individual accounts and broader patterns across the dataset [20].

Qualitative data from BRIS—anonymized chats and text messages from young people—were analyzed to capture both individual narratives and recurring patterns. BRIS provided anonymized logs from contacts registered within the predefined documentation category “Functional Impairments”. Preliminary analyses were conducted onsite using BRIS’s secure systems. All coding was performed locally, and no raw data was transferred outside BRIS. The method allows for a transparent, stepwise process where a priori questions are combined with inductively derived insights. The analytic process followed five stages: familiarization, identifying a thematic framework, indexing, charting, and mapping and interpretation.

### 2.2. Context and Data

Sweden is a Nordic country with approximately 10.6 million inhabitants. Although most young people report good general health, mental health problems such as anxiety, stress, and psychosomatic symptoms are relatively common among adolescents and have increased over recent decades, particularly among girls. This context is important for understanding young people’s help-seeking and experiences of mental ill-health in Sweden [22].

The dataset consisted of anonymized text-based contacts collected by BRIS during routine support activities in 2024, in which young people (approximately 10–18 years old) voluntarily initiated contact. The age range of approximately 10–18 years was based on ages explicitly reported by some young people within the contacts. Because the dataset was fully anonymised, detailed demographic information was not available to the research team. For this study, the year 2024 was selected because it represents a recent, fully completed operational year in which all chat-based support logs had been processed in accordance with GDPR and established data-management protocols. All material was generated through BRIS’s regular support services and analyzed in the original language, Swedish.

Quotations were translated from Swedish into English by bilingual members of the research team and reviewed by other bilingual authors. Minor linguistic adjustments were made for readability while preserving semantic meaning and emotional nuance. The original Swedish quotations are provided in Supplementary Table S3, and potential deductive-identification risks were considered before publication. The prefix C denotes chat contacts and S denotes text-message contacts. The identifiers were retained to enable linkage between quotations presented in the manuscript and the original Swedish excerpts provided in Supplementary Table S3. The identifiers are anonymised, nontraceable, and cannot be linked to individual users by either the research team or BRIS. The dataset consisted of complete anonymised chat conversations and text message contacts generated through BRIS’s routine support services. Both young people’s messages and counsellor responses were available within the original material. However, the analysis focused on young people’s own descriptions of the reasons for contacting BRIS (presenting problem).

All supportive contacts with the national helpline are documented according to 53 predefined support categories, each reflecting the child’s immediate help-seeking needs during the interaction. The category ‘Functional Impairments’ is routinely assigned by BRIS counsellors during or immediately after the support contact based on the young person’s expressed reason for seeking support. The categorisation is part of BRIS’s regular documentation procedures and was not conducted for research purposes. These categories do not represent background characteristics, nor should they be understood as diagnostic or mapping tools; rather, they are grounded in the child’s own stated needs at the time of contact. For the present study, the sampling frame was restricted to the documentation category “functional impairments”, defined as contacts in which young people sought support for diagnosed or suspected physical or neurodevelopmental conditions that affected their everyday life. The unit of analysis comprised young people’s own descriptions of the reasons for contacting BRIS (presenting problem), that is, how they articulated their support-seeking in their own words. The initial corpus comprised 126 chat conversations and 40 text messages, yielding a total of 166 contacts.

### 2.3. Sampling

A screening procedure based on predefined inclusion criteria was subsequently applied. Text contacts from 2024 were screened for relevance, and messages were included if young people referred to disabilities, diagnoses, or perceived functional challenges.

This approach made it possible to capture situations where health-related difficulties intersected with social and environmental contexts relevant to everyday functioning. To reduce the risk of including duplicate contacts, conversations with highly similar openings and content were removed during the selection process. However, due to the anonymised nature of the dataset, it was not possible to determine with certainty whether some contacts originated from the same individual at different time points.

No researcher-generated search terms were used. The sampling frame consisted of all contacts registered within the BRIS category “Functional Impairments”, one of 53 predefined support categories used within BRIS’s routine documentation system. The category is assigned by BRIS counsellors during or immediately after support contacts based on the young person’s expressed reason for seeking support and was not developed for research purposes. The BRIS documentation system assigns each contact to one primary support category. Consequently, contacts could not be registered in multiple support categories simultaneously.

### 2.4. Ethical Considerations

The study was based on anonymised routine service data provided by BRIS and analysed in accordance with the approved ethical procedures. Crisis- and suicide-related contacts were not excluded from the dataset. Any safeguarding procedures associated with such situations formed part of BRIS’s routine support practice at the time of the original contact and were managed by BRIS counsellors in accordance with organisational guidelines. The research team analysed historical anonymised data and therefore had no possibility of identifying, contacting, or intervening in individual cases. The ethical approval and the BRIS data-use agreement covered the use and publication of anonymised quotations. Young people were informed through the BRIS privacy framework that anonymised service data may be used for research and knowledge-development purposes.

### 2.5. Inclusion Criteria

Inclusion criteria were contacts from children and adolescents approximately 10–18 years of age, contact via BRIS (chat or text message), and an explicit reference to mental health challenges and associated stressors. Messages with low relevance to the study aim (e.g., technical inquiries or contacts not relevant to the study focus) were excluded according to the screening matrix. The category included contacts concerning both diagnosed and suspected conditions. As the focus of the study was on young people’s own descriptions and experiences, no distinction was made between confirmed and suspected diagnoses during the analysis. To be included in the analysis, contacts had to be registered within the Functional Impairments category and contain descriptions of challenges affecting mental health and/or everyday life that were relevant to the study aim. Contacts were excluded if they were assessed as having low relevance to the study aim or were identified as likely duplicates.

A total of 166 contacts (126 chat conversations and 40 text messages) were initially identified. During the screening process, 24 chat conversations and 5 text messages were excluded because they were assessed as having low relevance to the study aim or were identified as likely duplicates. The final analytic sample therefore consisted of 137 contacts (102 chat conversations and 35 text messages). Of the 29 excluded contacts, 20 were excluded because of low relevance and 9 because they were identified as likely duplicates. Low relevance referred to contacts not primarily concerning mental health challenges, functional difficulties, or issues relevant to the study aim. Screening was conducted by Nina Gårevik, Lise-Lott Rydström, and Petra Löfstedt, reviewed by Maria Jirwe, and uncertainties were resolved through discussion and consensus.

In accordance with the principle of information power [23], the research team judged that the dataset provided sufficient information to address the study aim. During analysis, the material became increasingly repetitive, and no substantially new codes, categories, or themes emerged.

### 2.6. Analytical Procedure

We used Framework Analysis [21], supported by manual coding in Excel and matrix-based organization. The full coding framework is provided in Supplementary Table S1. The analysis followed the Framework Analysis approach. The study aim and the focus on the young people’s reasons for contacting BRIS provided the starting point for the analysis. After becoming familiar with the material, the messages were coded based on their explicit content. Codes reflecting similar meanings were then grouped into subthemes derived from the young people’s narratives. The data were subsequently organised in an analytical matrix, allowing comparisons across contacts and categories. Each support contact constituted a case within the analytical matrix. Although binary indicators were used to organise the coded material, the analysis also incorporated free-text comments and repeated reference to the original contacts to preserve context and meaning. Codes with similar meanings were grouped into categories and subthemes, and the overarching theme was developed through iterative comparison and interpretation across cases.

Through an iterative process of comparison, synthesis, and interpretation, relationships between codes, subthemes, and overarching themes were identified. Coding was a dynamic process in which the initial codes were continuously reviewed and refined as a deeper understanding of the data developed and new insights emerged during the analysis. Likewise, subthemes and themes were continuously discussed within the research team and refined through repeated comparisons with the original data until a coherent interpretation was reached. Differences in interpretation were resolved through discussion and consensus. Throughout the analysis, the researchers repeatedly returned to the original messages to ensure that the interpretations remained grounded in the data. The coding framework and thematic structure are presented in the manuscript. The analytical matrix illustrating the relationships between codes, subthemes, and the overarching theme is provided in Supplementary Table S2. Illustrative examples showing how raw text excerpts contributed to code development, subthemes, and the overarching theme are provided in Supplementary Table S3.

### 2.7. Manual Coding Process

The coding process was conducted manually. Familiarisation with the data was undertaken by NG, LLR, and PL through repeated reading of the contacts. The preliminary analytical framework was developed by NG and MJ. Primary coding, data management, and charting within the analytical matrix were undertaken by NG. Interpretation and refinement of codes, categories, subthemes, and themes were conducted by NG and MJ and discussed with LLR and PL. No independent double-coding of any subset of the material was undertaken. Differences in interpretation were discussed throughout the process until consensus was reached. No automated or software-based coding tools were used.

Throughout the analytical process, interpretations and emerging themes were continuously discussed within the research team. Repeated comparisons between the original data, codes, and thematic structure were conducted to ensure that the findings remained grounded in the young people’s accounts. The analytical process was documented throughout to support transparency and consistency in the interpretation of the data. Variation within the data was actively considered throughout the analytical process. Although young people described different circumstances and experiences, the data consistently reflected the patterns captured by the overarching theme and subthemes. No sufficiently distinct patterns emerged that warranted the development of additional themes.

The research team included researchers with backgrounds in psychiatric nursing, paediatric nursing, paediatric intensive care nursing, public health, and qualitative health research. None of the researchers were employed by or affiliated with BRIS. Analytical notes were documented during the coding process to support reflection and consistency in the interpretation of the data. Several members of the research team had experience in nursing research and in working with children, adolescents, and mental health-related issues. Throughout the analysis, the researchers reflected on how their professional backgrounds might influence the interpretation of the data.

Credibility was enhanced through repeated comparisons between the original data and the evolving coding structure, as well as the ongoing refinement of interpretations. Coding decisions and analytical reflections were documented, and the coding matrix was revised as the work progressed. Regular discussions within the research team helped ensure a shared understanding of the data and contributed to a transparent and dependable analytical process.

## 3. Results

The analysis identified one overarching theme: everyday life was often described as a constant effort to manage emotional strain. Three subthemes capture how this was expressed in social contexts, at school, and within the family. The overarching theme, “Trying to Survive in an Emotional Storm”, was developed during the interpretative phase of the analysis as a synthesis of the patterns identified across the subthemes. It was intended to capture the shared experience reflected across young people’s accounts. The quotations presented below were selected to illustrate central patterns in the data while also reflecting variation in young people’s experiences across the subthemes.

### 3.1. Overarching Theme: Trying to Survive in an Emotional Storm

Across the accounts, everyday life was portrayed as demanding and at times difficult to manage. Feelings of fear, uncertainty, and a sense of not coping recurred and interacted with experiences of loneliness, family conflict, and school-related pressure. Taken together, the young people’s descriptions suggest that this is not a matter of isolated problems, but rather an ongoing struggle with situations perceived as overwhelming and difficult to influence. Ways of coping varied. Some sought support, others withdrew from social situations, and in some cases, strategies could be harmful. These situations often occurred in contexts described as unpredictable or characterized by a lack of support. At the same time, both vulnerability and a strong effort to make everyday life work emerge, despite the difficult and often unpredictable circumstances described by the young people. Together, this highlights how everyday life is shaped by a continuous effort to manage a complex and hard-to-influence life situation.

### 3.2. Subtheme: Social Environment—Between Norm Pressure and the Need for Support

The emotional strain described in overarching Theme 1 is particularly visible in social environments, where the accounts reveal strong norms related to behaviour, performance, and social competence. Many describe not fitting in at school or among peers, leading to feelings of exclusion, shame, and loneliness. Norms related to social interaction, emotional regulation, and adaptation often appear difficult to meet, particularly among young people describing neurodevelopmental conditions or mental health difficulties. References to ADHD, sometimes described as ADD (attention-deficit disorder), also emerge in the narratives as a way of understanding social difficulties and experiences of rejection. Taken together, these descriptions suggest that the difficulties are not solely related to individual circumstances, but also to navigating social expectations that are experienced as difficult to fulfil. One young person expressed this as follows:

*“I have ADHD and I hate it because every time I try to get a friend, they don’t like me”*

(S143888)

Similarly, another account illustrates how perceived difference becomes a source of stigma:

*“I have no friends in school. I have ADD. I’m ashamed of how different I am from the others”*

(C261544)

Across these accounts, diagnoses are used as a way of making sense of experiences of exclusion in social contexts shaped by normative expectations. Social pressure is also described as being reinforced by adults in school settings, particularly when behaviours are interpreted as disruptive rather than as expressions of a need for support:

*“My teacher just screams at me because I can’t sit still”*

(C254619)

Such responses are described as contributing to feelings of exclusion and being misunderstood.

At the same time, a strong desire for supportive relationships with both peers and adults who listen and show understanding emerges. When such support is lacking, loneliness and despair become particularly prominent:

*“I feel so incredibly lonely it almost kills me. I have no one to talk to, I have no friends, I’m just bullied at school”*

(C2500988)

However, even limited forms of support may be experienced as meaningful:

*“I have a support person at school, talk to him sometimes. I don’t like talking to mom and dad”*

(C267503)

Being listened to and understood emerges as particularly important in the accounts. Even limited support may offer a sense of connection and reduce feelings of loneliness in otherwise challenging circumstances. Such relationships appear to provide a space where difficulties can be shared and acknowledged, rather than faced alone.

### 3.3. Subtheme: A Constant Struggle in the Educational System

The emotional strain described in Theme 1 is also evident within the educational system, where school is often portrayed as a setting characterised by stress and emotional burden. The accounts describe high demands, insufficient support, and a sense that others do not take their difficulties seriously. Several express self-criticism and a feeling of being unable to meet expectations:

*“My grades are only E’s and some F’s… I cry in school almost every day… no one understands me”*

(C266949)

Some accounts describe how promised support fails to result in any meaningful change:

*“We were supposed to get accommodations, but it was just empty words; all I get is to sit in the back”*

(C249445)

School-related difficulties are frequently linked to anxiety, particularly when learning difficulties or neurodevelopmental conditions form part of the situation:

*“I have an F in math. I have dyslexia and ADHD… I get extreme anxiety because of math”*

(S142847)

Beyond diagnoses, the accounts describe a broader pattern in which young people often experience themselves as carrying the primary responsibility for managing school-related difficulties. When support is perceived as insufficient or absent, a sense of having to cope with these challenges alone emerges. This appears to contribute to stress, school absenteeism, and hopelessness, as difficulties perceived to be beyond one’s control simultaneously become something the individual is expected to manage. Some accounts described concerns related to medication use, including perceived changes in well-being, daily functioning, and school experiences. One young person described substantial weight loss while also expressing concern about discontinuing medication because of perceived benefits for school performance:

*“I have ADD… I’ve lost 23 kilos since I started medication… I’ve finally managed to improve my grades, and I don’t want to stop taking my medication now”*

(S144783)

Others express frustration in their contacts with healthcare in relation to school-related stress, particularly when support is experienced as consisting primarily of medication adjustments without sufficient information or explanation:

*“All they do is prescribe new medicine. They don’t explain how it is supposed to help or what side effects it may have”*

(C258117)

The accounts illustrate how school difficulties, mental health problems, and experiences of insufficient support form an interconnected experience of vulnerability, characterised by uncertainty and limited opportunities to influence one’s own situation.

### 3.4. Subtheme: Insufficient Parental Support—Between Desire for Help and Barriers to Understanding

The emotional strain described in Theme 1 is also reflected in relationships with parents, where difficulties in receiving understanding and support were common across the accounts. When young people tried to seek help, they often described responses such as disbelief or minimization, and in some cases concerns about what a diagnosis might mean:

*“I think I have ADHD, but my mom doesn’t want to start an assessment”*

(C247046)

Some accounts also show how young people felt that their experiences were dismissed or not taken seriously:

*“My parents say I don’t have it… but I know something is wrong”*

(S142940)

In other cases, resistance from parents was linked to worries about the consequences of a diagnosis:

*“My dad says he doesn’t want me to have a diagnosis because it will stop me from doing certain things”*

(C285414)

The material also includes descriptions of family conflict, emotional insecurity, and limited communication. For some, this appeared to make it more difficult to access professional support. In several accounts, a lack of understanding seemed to limit opportunities for shared understanding and support within the family. It also added to the emotional strain, as many described trying to manage their situation on their own.

A recurring pattern across the accounts was that attempts to seek support were often met with disbelief, minimization, or concerns about diagnoses rather than the understanding being sought. This appeared to restrict opportunities for support within the family while increasing the burden of managing difficulties alone. Taken together, these accounts suggest that limited parental understanding may intensify the emotional storm described in the overarching theme, leaving young people with fewer resources to cope with overwhelming everyday demands.

## 4. Discussion

This study identified the overarching theme *Trying to Survive in an Emotional Storm*, reflecting how young people described everyday life as emotionally demanding and difficult to manage. The three subthemes illustrated how these experiences were shaped by social relationships, school, and family contexts. The analysis of anonymous youth helpline contacts provides insight into how young people themselves describe these challenges when seeking support.

Although neurodevelopmental difficulties were frequently mentioned, the analysis focused on how young people themselves understood and described their experiences rather than on diagnostic categories. The findings highlight emotional strain, experiences of exclusion, unmet support needs, and the use of diagnostic language as a way of making sense of difficulties.

These findings can be understood through Self-Determination Theory, which describes autonomy, competence, and relatedness as central to well-being [15,16,24]. Previous research shows that being listened to with attention and understanding, without being judged, can make a meaningful difference. Such listening may enhance well-being, particularly by strengthening a sense of belonging [17]. From a nursing perspective, particularly that of school nurses, it becomes clear that a responsive and respectful approach is essential in encounters with young people. School nurses are often among the first professionals that young people encounter when seeking support and therefore play an important role in promoting health and identifying support needs at an early stage [4]. Within person-centred care, this involves healthcare professionals engaging with and seeking to understand the individual’s narrative as a basis for care [10]. For nurses, this means creating space for young people’s voices and acknowledging their experiences.

Studies suggest that young people need to be seen as active agents in their own lives, rather than merely as objects of assessment [25]. This is consistent with a child-centred approach to nursing, in which the child’s voice and experiences are given a central role, and where adult dominance may otherwise limit participation [26]. Previous research, primarily focusing on children but also relevant to adolescents, shows that opportunities for participation in healthcare are largely shaped by the conditions created by adults, and that factors such as parental influence or a failure to take the child’s needs into account can restrict participation [27]. When such conditions are lacking, young people’s experiences of care may instead be characterized by limited autonomy and reduced influence over decisions that affect them. These patterns are consistent with prior research on psychiatric interventions. Studies of adolescents’ experiences of medication can be seen as an example of how treatment may at times be perceived as a threat to autonomy and identity, particularly when decision making is driven by adults [28]. Comparable findings have also been described in previous research, including in adult populations, where biomedical ways of understanding distress may be experienced as limiting or stigmatizing [29].

Viewed through the lens of the International Classification of Functioning, Disability and Health (ICF), the findings show that young people’s functioning is shaped through interaction with their environment, with young people seeking support on their own terms rather than being understood solely in terms of their difficulties [2].

Research shows that young people’s relationships with psychiatric diagnoses are complex and not solely about accepting a diagnosis, but also about the opportunity to express their own narratives without being defined by it [30]. Studies of young people further indicate that they often have limited influence over treatment decisions and that family involvement may shape these decisions, underscoring the importance of autonomy in care [31]. Overall, this highlights the importance of an approach that gives space to young people’s own perspectives and where care is characterized by participation and collaboration [30,31]. However, young people’s participation is often deprioritized in practice due to time and resource constraints [25], while adult-driven decisions may limit autonomy [32]. Clinical guidelines emphasize shared decision making and provider responsibility [33], highlighting the need for practices that actively support young people’s participation and autonomy. In line with international norms on inclusive education, the right to education entails appropriate adjustments and shared responsibility, underscoring that support cannot be placed solely on the student [34]. This is consistent with our findings, which show that support needs to be adapted to young people’s life situations rather than being directed solely at the individual.

Studies further show that adolescents, for example, those with ADHD, experience the diagnosis as both helpful and challenging: it can support understanding, but also carries risks of stigma. As such, the diagnosis may function as a resource for meaning-making and autonomy, while also contributing to marginalization [35,36,37]. The present findings suggest a similarly nuanced picture. Diagnostic terminology may serve different functions for young people and should not be understood solely as an expression of a medical diagnosis. For some, diagnostic labels may represent a way of understanding themselves and their experiences, while for others they may provide a socially recognised language for describing emotional distress and difficulties in everyday life. The findings do not allow conclusions about the specific meaning that diagnostic terms held for individual young people. Rather, they indicate that diagnostic language may be one of several ways in which young people interpret, understand, and communicate their experiences.

From a nursing perspective, this means that healthcare professionals need to work in ways that support young people’s participation and autonomy, and that are grounded in their own experiences and narratives. This is also supported by research showing that children’s mental health is shaped through interaction with their environment, where factors such as participation, relationships, and family context are of importance [38]. This underlines the need for support that is directed not only at the individual, but also at the contexts in which young people live.

### 4.1. Implications for Practice, Education and Future Research

Going forward, care needs to move beyond a narrow focus on diagnoses and instead work to strengthen young people’s functioning in everyday life through individualized support, early interventions, and collaboration with schools and families. From a nursing perspective, this involves supporting participation, autonomy, and manageability in everyday life, as well as involving the family in the care process. For school nurses, this may involve actively exploring how difficulties in everyday life affect school participation and well-being, rather than focusing solely on diagnoses or symptoms. For nurses working in child and adolescent mental health services, the findings highlight the importance of involving young people in discussions and decisions about interventions and support. This may also involve supporting young people’s understanding of themselves and their experiences alongside, rather than solely through, diagnostic labels. Paediatric nurses may similarly benefit from exploring how health-related difficulties affect young people’s everyday lives, relationships, and opportunities for participation. Across settings, nurses can support young people by creating opportunities for them to describe their experiences in their own words and by recognising the meanings they attach to diagnostic language and support needs. At the same time, a potential stigmatization paradox needs to be acknowledged, whereby young people’s use of diagnostic language to understand and express their experiences may risk leading to misunderstandings if this language is not recognized in care encounters.

Future research should further explore how person-centred and family-oriented approaches can be implemented in different care settings, and how young people’s participation and use of diagnostic language can be better integrated into care encounters. In addition, there is a need to explore how young people can be supported in developing self-understanding beyond diagnostic frameworks, in ways that promote autonomy and meaning-making. Participatory approaches involving young people would be valuable, as would research examining how youth helpline support interacts with support from families, schools, and healthcare services.

### 4.2. Strengths and Limitations

A strength of this study lies in the access to young people’s own narratives within an anonymous context, enabling the collection of experiences that often do not emerge in care settings. The analysis has allowed for a systematic and close interpretation of these narratives, which strengthens the credibility of the study from a nursing perspective. At the same time, the findings highlight how young people who use diagnoses to make sense of their suffering may become a site where different epistemological perspectives intersect and thereby risk once again being caught in an “emotional storm,” where differing understandings within healthcare may reinforce rather than alleviate their situation. Finally, it is important to understand the significance that diagnoses may have for young people, while at the same time ensuring that care considers the overall context of their lives and the different challenges they face across various settings.

This study has some limitations inherent to qualitative, text-based research. The data consist of written chat and text messages, which limit access to paralinguistic cues such as tone, pauses, body language, and emotional expression that may be present in face-to-face interactions. A further limitation of the study is that the data consisted of naturally occurring support conversations rather than research interviews. Consequently, it was not possible to ask follow-up questions or clarify ambiguous statements, which may have limited the depth of some accounts.

In addition, the narratives reflect a specific support context, where the way the service is organized, documented, and delivered may have influenced how young people expressed their distress and need for support. As with all qualitative research, the findings are contextually situated and analytically interpreted rather than intended for statistical generalization. The results should therefore be understood as offering in-depth insight into the experiences of young people who contact BRIS for support, rather than as representative of all adolescents with mental health difficulties.

Participants were self-selected and may overrepresent young people experiencing acute distress or specific concerns. The findings should therefore be interpreted in relation to this context rather than generalized to all young people or help-seeking situations. The data consisted of text-based contacts collected during 2024 and reflects the experiences of young people who actively chose to seek support through BRIS. Consequently, the findings should not be interpreted as representative of all young people or clinical populations.

Furthermore, information regarding young people’s gender was not available in the anonymised dataset. Consequently, potential similarities and differences in how young people described mental health challenges, functional difficulties, and support needs could not be explored. The prominence of ADHD/ADD and school-related distress in the findings reflects recurring topics within the analysed material rather than a predefined analytical focus or selection procedure.

Restricting the sampling frame to the administrative category “Functional Impairments” may have introduced classification and selection bias. It is possible that relevant contacts concerning functional difficulties were recorded under other BRIS support categories and were therefore not included in the present study. The findings should also be understood in relation to the Swedish context and the specific role of BRIS as a national support service for children and adolescents. Young people’s experiences of support-seeking may be influenced by the organisation of healthcare, educational, and welfare systems, which vary across countries. Transferability should therefore be considered in relation to similar contexts and support services.

Young people were not involved in the design, analysis, interpretation, or dissemination of the study. Future research could benefit from greater involvement of young people throughout the research process. Although anonymization was rigorous and the analysis iterative and reflexive, the dataset remains context-bound. Supplementary Materials are provided to support transparency.

## 5. Conclusions

In conclusion, this study identified patterns in how young people contacting a national youth helpline described challenges affecting their mental health and everyday lives. The findings suggest that relational and contextual factors were important in how distress was experienced and understood. They also indicate that diagnostic language may play different roles in how young people make sense of and communicate their difficulties. For nurses, the key message is the importance of listening to and working from young people’s own narratives and understandings of their difficulties, rather than focusing solely on diagnoses or symptoms.

## Data Availability

The data used in this study consist of confidential anonymized support contacts provided by BRIS (Children’s Rights in Society) and are not publicly available. Access to the data is subject to BRIS governance procedures, confidentiality requirements, and applicable ethical approvals. Requests for access should therefore be directed to BRIS and will be considered in accordance with relevant legal, ethical, and organizational regulations.

## Supplementary Materials

The following supporting information can be downloaded at: https://www.mdpi.com/article/10.3390/nursrep16080284/s1, Table S1: Coding Framework. Steps in Framework Analysis and Their Operationalization in the Study; Table S2: Analytical matrix illustrating the relationships between codes, subthemes, and the overarching theme; Table S3: Illustrative examples of the analytical process from code to theme.

## Public Involvement Statement

No public involvement in any aspect of this research.

## Guidelines and Standards Statement

This manuscript was prepared in accordance with the Consolidated Criteria for Reporting Qualitative Research (COREQ) guidelines for qualitative research [39].

## Abbreviations

The following abbreviations are used in this manuscript:

ADHD	Attention-Deficit/Hyperactivity Disorder
ADD	Attention-Deficit Disorder
BRIS	Children’s Rights in Society
ICF	International Classification of Functioning, Disability and Health
SDT	Self-Determination Theory
