# Supplementary material for: Trying to Survive in an Emotional Storm: A Qualitative Framework Analysis of Anonymous Youth Helpline Conversations on Mental Health and Everyday Life
Source: Nurs Rep. 2026 Aug 15;16(8):284. doi: 10.3390/nursrep16080284 (PMC13516053; doi:10.3390/nursrep16080284)
Supplement: Supplementary file 1 [file nursrep-16-00284-s001.zip › nursrep-4466711-supplementary.pdf]

Table S1: Coding Framework. Steps in Framework Analysis and Their Operationalization in the Study

**Step (Framework Analysis)**	**Purpose**	**Implementation in This Study**	**Analytical Product**
1. Familiarization	Holistic understanding of the material	Read anonymized chats and SMS focusing on young people's reasons for contacting BRIS and challenges affecting mental health and everyday life; note recurring expressions (e.g., school pressure, diagnosis, loneliness).	Initial notes and a preliminary list of codes focused on reasons for contact and context.
2. Identifying a Framework	Develop a manageable coding structure	Combine **predefined analytical interests** (presenting problem, mental health, diagnosis) with inductively emerging categories; define codes through iterative comparison with the data.	Preliminary coding framework with code, category, and subtheme definitions.
3. Indexing (Coding)	Systematic assignment of text to codes	Coding involved the use of binary indicators (1/0) to organise patterns across contacts, together with free-text comments capturing context, meaning, and illustrative excerpts. Multiple codes could be assigned to the same contact when relevant.	Coded data matrix (ID × codes) with contextual comments.
4. Charting (Matrixing)	Summarize and organize data thematically	Produce condensed summaries per category and subtheme; enable comparisons across contacts and communication channels (chat/SMS).	Thematic charts supporting cross-sectional and case-based views.
5. Mapping & Interpretation	Identify patterns, relationships, and meaning	Integrate matrices with original excerpts to derive subthemes and the overarching theme; iteratively validate interpretations against source text.	Overarching theme and subthemes presented in the Results section.

Table S2: Analytical matrix illustrating the relationships between codes, subthemes, and the overarching theme.

Code Column (Matrix)	Overarching Theme	Subtheme
Social isolation and bullying	Trying to Survive in an Emotional Storm	Social Environment – Between Norm Pressure and the Need for Support
Normative constraints (performance, behavior)	Trying to Survive in an Emotional Storm	Social Environment – Between Norm Pressure and the Need for Support
Feeling different / stigma	Trying to Survive in an Emotional Storm	Social Environment – Between Norm Pressure and the Need for Support
Peer rejection	Trying to Survive in an Emotional Storm	Social Environment – Between Norm Pressure and the Need for Support
Need for support but lacking access	Trying to Survive in an Emotional Storm	Social Environment – Between Norm Pressure and the Need for Support
School-related stress	Trying to Survive in an Emotional Storm	Constant Struggle in the Educational System
High performance demands	Trying to Survive in an Emotional Storm	Constant Struggle in the Educational System
Insufficient accommodations	Trying to Survive in an Emotional Storm	Constant Struggle in the Educational System
Not being believed by teachers	Trying to Survive in an Emotional Storm	Constant Struggle in the Educational System
Academic anxiety / feeling like a failure	Trying to Survive in an Emotional Storm	Constant Struggle in the Educational System
Medication affects school functioning	Trying to Survive in an Emotional Storm	Constant Struggle in the Educational System

Difficulty accessing assessment	Trying to Survive in an Emotional Storm	Insufficient Parental Support – Between Desire for Help and Barriers to Understanding
Parental disbelief / minimization	Trying to Survive in an Emotional Storm	Insufficient Parental Support – Between Desire for Help and Barriers to Understanding
Conflict at home around diagnosis	Trying to Survive in an Emotional Storm	Insufficient Parental Support – Between Desire for Help and Barriers to Understanding
Worry about tensions at home	Trying to Survive in an Emotional Storm	Insufficient Parental Support – Between Desire for Help and Barriers to Understanding
Lack of parental understanding	Trying to Survive in an Emotional Storm	Insufficient Parental Support – Between Desire for Help and Barriers to Understanding

Table S3. Illustrative examples of the analytical process from code to theme

Raw text excerpt	Code	Subtheme	Overarching theme
"Jag är ganska säker på att jag har ADHD ... jag vill ta reda på det."	Diagnostic uncertainty	Insufficient Parental Support – Between Desire for Help and Barriers to Understanding	Trying to Survive in an Emotional Storm
"Det är ofantligt mycket arbete i skolan, jag blir besviken om jag inte når A-nivå."	School-related stress	Constant Struggle in the Educational System	Trying to Survive in an Emotional Storm
"Mina föräldrar säger att jag inte har det."	Parental disbelief/minimization	Insufficient Parental Support – Between Desire for Help and Barriers to Understanding	Trying to Survive in an Emotional Storm
